# The wellbeing neuro course: a randomised controlled trial of an internet-delivered transdiagnostic psychological intervention for adults with neurological disorders

**DOI:** 10.1017/S0033291723000338

**Published:** 2023-10

**Authors:** Milena Gandy, Andreea I. Heriseanu, Tanya Balakumar, Eyal Karin, Jennie Walker, Taylor Hathway, Madelyne A. Bisby, Amelia J. Scott, Joanne Dudeney, Alana Fisher, Nickolai Titov, Blake F. Dear

**Affiliations:** 1 eCentreClinic, School of Psychological Sciences, Macquarie University, Sydney, Australia; 2 MindSpot Clinic, Macquarie University, Sydney, Australia

**Keywords:** Anxiety, cognitive behaviour therapy, depression, digital, neurology, psychotherapy

## Abstract

**Background:**

Mental health and functional difficulties are highly comorbid across neurological disorders, but supportive care options are limited. This randomised controlled trial assessed the efficacy of a novel transdiagnostic internet-delivered psychological intervention for adults with neurological disorders.

**Methods:**

221 participants with a confirmed diagnosis of epilepsy, multiple sclerosis, Parkinson's disease, or an acquired brain injury were allocated to either an immediate treatment group (*n* = 115) or treatment-as-usual waitlist control (*n* = 106). The intervention, the Wellbeing Neuro Course, was delivered online via the eCentreClinic website. The Course includes six lessons, based on cognitive behavioural therapy, delivered over 10 weeks with support from a psychologist via email and telephone. Primary outcomes were symptoms of depression (PHQ-9), anxiety (GAD-7) and disability (WHODAS 2.0).

**Results:**

215 participants commenced the trial (treatment *n* = 111; control *n* = 104) and were included in intention-to-treat analysis. At post-treatment, we observed significant between-group differences in depression (PHQ-9; difference = 3.07 [95% CI 2.04–4.11], *g* = 0.62), anxiety (GAD-7; difference = 1.87 [0.92–2.81], *g* = 0.41) and disability (WHODAS 2.0 difference = 3.08 [1.09–5.06], *g* = 0.31), that favoured treatment (all ps < 0.001). Treatment-related effects were maintained at 3-month follow-up. Findings were achieved with minimal clinician time (average of 95.7 min [s.d. = 59.3] per participant), highlighting the public health potential of this approach to care. No adverse treatment events were reported.

**Conclusions:**

Internet-delivered psychological interventions could be a suitable model of accessible supportive care for patients with neurological disorders.

Depression and anxiety are highly prevalent among people with neurological disorders (Hesdorffer, 2016) and substantially increase burden of disease. Poor mental health can disrupt the medical management, self-management, quality of life, and prognosis of neurological disorders (Gandy et al., 2021; Hesdorffer, 2016; Patel et al., 2016). In addition, neurological disorders are often associated with significant disability, especially difficulties with cognitive function. Unfortunately the mental health and rehabilitation needs of neurology patients remains an area of considerable unmet need (Hesdorffer, 2016; WHO, 2020) with the World Health Assembly recently emphasising the importance of reform in this area via the Intersectoral Global Action Plan on Epilepsy and other Neurological Disorders (IGAP) (WHO, 2020, 2022). Moreover, the integration of mental health and other supportive care is largely absent in neurology (Chan, Toccalino, Omar, Shah, & Colantonio, 2022; Creutzfeldt et al., 2018) and lags behind the integrated care models used to manage other chronic health conditions (Butow, Dhillon, Shaw, & Price, 2017; Kohrt, Griffith, & Patel, 2018).

Evidence demonstrates that mental health outcomes can be improved via psychological interventions, such as cognitive behaviour therapy (CBT), in the general population (Butler, Chapman, Forman, & Beck, 2006) and there is emerging evidence for CBT in neurological disorders (Fernie, Kollmann, & Brown, 2015; Waldron, Casserly, & O'Sullivan, 2013). Additionally, such interventions can assist patients manage comorbid day-to-day functional difficulties (e.g. fatigue and memory) (Chalah & Ayache, 2018; Radford, Lah, Thayer, Say, & Miller, 2012). Nevertheless, there are significant barriers including costs, lack of trained specialists, and challenges with mobility, that prevent access to these interventions (Gandy et al., 2018).

Psychological interventions delivered via the Internet are a recent paradigm-shifting development that are improving access to psychological therapies including CBT (known as iCBT) (Andersson, Titov, Dear, Rozental, & Carlbring, 2019). These interventions teach the same psychological skills as face-to-face treatments but use carefully developed online modules often with clinician support via telephone and email. Substantial research now supports the feasibility and efficacy of iCBT and their clinical equivalence with face-to-face care in the general population (Carlbring, Andersson, Cuijpers, Riper, & Hedman-Lagerlöf, 2018). Research also indicates these interventions are clinically efficacious for patients with chronic physical health conditions (Mehta, Peynenburg, & Hadjistavropoulos, 2019).

Despite their significant potential, less is known about the efficacy of internet-delivered psychological interventions for common neurological disorders. The few available studies of iCBT in neurological disorders have generated promising results (Fischer et al., 2015; Kraepelien et al., 2020; Meyer et al., 2019; Schröder et al., 2014) but are limited to small-sized early phase trials. Studies to date have also utilised disorder-specific protocols targeting only one neurological disorder (e.g. epilepsy) and only one domain of mental health or functional outcome (e.g. depression only). To increase the scope and reach of iCBT for neurology patients, we recently conducted a Phase I open trial of a novel transdiagnostic internet-delivered psychological intervention, the Wellbeing Neuro Course (Gandy et al., 2020). This programme aims to teach adults with a variety of neurological disorders psychological skills to manage both their mental health and functional difficulties. We trialled this intervention in 105 people with either a diagnosis of multiple sclerosis (MS), epilepsy, Parkinson's disease (PD) or an acquired brain injury (ABI), where there is already some evidence for the safety and efficacy of CBT (Fernie et al., 2015; Waldron et al., 2013). Our feasibility trial found high levels of acceptability and significant moderate to large within-group effects for primary outcomes of depression, anxiety and disability and small effects for secondary outcomes of cognitive difficulties and fatigue (Gandy et al., 2020).

In the current study we examined the acceptability and efficacy of the Wellbeing Neuro Course via a two-group randomised controlled trial (RCT). The study sought to determine whether participants in the intervention reported significant improvements in symptoms of depression, anxiety, and disability immediately post-treatment when compared to a treatment-as-usual waitlist control (TAU-WLC) group, and whether improvements were maintained at 3-month follow-up.

## Method

### Participants

The RCT was conducted via a specialist research clinic, the eCentreClinic, at Macquarie University, Sydney, Australia. Prospective participants read details of the trial and made an application via the eCentreClinic website (www.ecentreclinic.org). Previous consenting participants of an online national mental health survey (Gandy et al., 2018) were also sent details of the trial and the course was advertised via the eCentreClinic Facebook page and several Australian neurology advocacy groups.

Participants were eligible if they met the inclusion criteria: (1) Australian resident, (2) ≥18 years, (3) formal diagnosis of epilepsy, MS, PD, or ABI, and (4) reported that their disorder affected their emotional and/or cognitive health. Exclusion criteria were: (1) an inability to access or use a computer and the Internet, (2) very severe depression symptoms indicated by a total score >25 on the Patient Health Questionnaire 9-item (PHQ-9) (Kroenke, Spitzer, & Williams, 2001), (3) significant suicidal ideation (i.e. a score >2 to Question 9 on the PHQ-9) (Kroenke et al., 2001) or acute suicidality or recent suicide attempt (i.e. last 12-months), and (4) serious cognitive impairment (<21 on the Telephone Interview of Cognitive Status; TICS) (Brandt et al., 1993). Given the transdiagnostic nature of the intervention, no minimal symptom limits were imposed on the primary outcomes at study entry but are accounted for in sensitivity analyses.

All consenting participants completed an online screening assessment. Eligible participants were then contacted by study clinicians to further assess eligibility and describe the study. Participants provided details of their treating general practitioner and/or neurologist, who were faxed a letter notifying them of their patient's participation, inviting contact should they have any questions or concerns, and requesting confirmation of their patient's diagnosis.

### Randomisation

Randomisation was performed by an independent researcher using an online randomiser (www.random.org) using permuted blocks of 6, with 1:1 allocation ratio stratified based on the primary neurological disorder. The allocation sequence was concealed from investigators until participants were successfully enrolled. Participants randomised to the treatment group commenced the 10-week intervention period, after which time those allocated to the TAU-WLC group commenced treatment. This study was approved by the Human Research Ethics Committee of Macquarie University and was prospectively registered ACTRN12620000165987 without trial changes.

### Procedures

The Wellbeing Neuro Course is an internet- delivered psychological intervention which integrates principles of CBT and compensatory cognitive rehabilitation therapy to teach therapeutic skills to address the common mental health and functional impacts of neurological disorders. The intervention is based on transdiagnostic treatment model, to target several domains of wellbeing, and to allow broad applicability across different neurological disorders. The intervention provides the same psychological skills as face-to-face treatments but delivers this information via carefully developed online modules and remote clinician support. A comprehensive overview of the intervention is provided in online Supplementary materials (Supplementary Table S1) and reported elsewhere (Gandy et al., 2020). The intervention includes guidance on how to adopt psychological skills, including three comprehensive case stories of adults with neurological disorders. It was carefully designed to minimise cognitive overload and facilitate engagement. Participants worked through the course according to a predetermined timetable and could not access new materials without first having read previous materials. The intervention was delivered in conjunction with clinical contact from a psychologist with specialist training in clinical psychology (MG, AH, TB) via weekly telephone calls and/or emails delivered via a secure messaging system. The primary purpose of clinician contact was to support and encourage participants to work through the Wellbeing Neuro Course and to guide participants in the application of the skills in the context of their unique symptoms and circumstances.

### Outcome measures

Prespecified primary outcome included self-reported symptoms of depression, anxiety and disability. Depression was assessed using the widely-used and validated PHQ-9 (Kroenke et al., 2001) with a score of >9 indicative of clinically significant symptoms. Anxiety was assessed using the well-validated Generalised Anxiety Disorder Scale 7-Item (GAD-7) (Spitzer, Kroenke, Williams, & Löwe, 2006) with a total score of >9 considered the clinical range (Löwe et al., 2008). Disability was assessed using the 12-item World Health Organization Disability Assessment Schedule 2.0 (WHODAS 2.0) (Ustun et al., 2010), which assesses functioning across self-care, communication, mobility, interpersonal relationships, life activities, and community participation, with a score >9 considered clinically significant (Andrews, Kemp, Sunderland, von Korff, & Ustun, 2009).

Secondary outcomes included three subscales of the Neuro-QoL (Quality of Life in Neurological Disorders) (Cella et al., 2012) measurement system. This included the 8-item Cognitive Function scale (e.g. perceived difficulties with memory), the 8-item Emotional and Behavioural Dyscontrol scale (e.g. symptoms of irritability) and 9-item Positive Affect and Wellbeing (e.g. life satisfaction). The use of everyday cognitive strategies (e.g. diary use) was assessed using a purpose built Compensatory Cognitive Strategies Questionnaire (CCSQ) (see online Supplementary Methods 1).

At post-treatment, intervention satisfaction and acceptability were assessed via a 3-item purpose-built questionnaire, as described elsewhere (Gandy et al., 2020).

For validation purposes two neurological specific measures of symptoms of depression (Neurological Depressive Disorders Inventory-Epilepsy; NDDI-E) (Gilliam et al., 2006) and anxiety (The Brief Epilepsy Anxiety Symptom Inventory; brEASI) (Scott et al., 2019) were administered. These measures were designed to remove items that may be confounded by neurological phenomena (e.g. seizures) and side effects of medication. Findings related to these measures are included as online Supplementary files (Supplementary Tables S4 and S5).

### Statistical analysis

Sample size was determined employing data from the Phase 1 trial (Gandy et al., 2020), which indicated a sample with 100 per group (*N* = 200) is powered to detect differences between groups that are as small as 11% on the primary outcomes with alpha set at 0.05 and power set at 0.80.

Analysis was conducted in IBM SPSS version 28. Descriptive statistics were calculated regarding participants’ demographic and baseline clinical characteristics (Table 1). No statistically significant baseline differences were observed between the treatment and control groups on any participant characteristics, including symptom severity on outcomes measures (*p* values >0.05). Efficacy analyses used an intent-to-treat approach, where all randomised participants who provided baseline data were represented at all time points in the relevant analyses. To address missing data (only 5% at post-treatment and 11% at 3-month follow-up), a multiple imputation procedure was applied. The multiple imputation model included participants’ neurological disorder type, baseline symptom severity, and the number of treatment lessons completed. Both baseline symptom severity and lesson completion have been identified as important and non-ignorable mechanisms of missing data (Karin, Dear, Heller, Crane, & Titov, 2018a).

**Table 1.** Baseline participant demographic and clinical characteristics

	Treatment (*n* = 111)	Control (*n* = 104)
	*N* (%)	*N* (%)
Sex		
Female	91 (82)	79 (76)
Male	20 (18)	24 (23)
Other	0	1 (1)
Age (Mean years [s.d.; Range])	53 (12; 25–79)	53 (13; 27–81)
≤29	3 (3)	3 (3)
30–39 years	14 (13)	14 (13)
40–49 years	24 (22)	28 (27)
50–59 years	38 (34)	28 (27)
60–69 years	22 (20)	18 (17)
70–79 years	10 (9)	12 (12)
≥80	0	1 (1)
Employment^a^		
Current employment	46 (41)	39 (38)
Unemployed	8 (7)	14 (14)
Registered disability	27 (24)	23 (22)
Retired	31 (28)	27 (26)
In current relationship^b^		
Yes	72 (65)	63 (61)
No	39 (35)	41 (40)
Education		
High school	20 (18)	25 (24)
Trade certificate	12 (11)	16 (15)
Undergraduate/ associate diploma	30 (27)	23 (22)
Higher research degree	49 (44)	40 (38)
Primary Neurological Disorder^c^		
Multiple Sclerosis	41 (37)	44 (42)
Acquired Brain Injury^d^	29 (26)	24 (23)
Parkinson's disease	25 (23)	18 (17)
Epilepsy	16 (14)	18 (17)
Comorbid targeted neurological disorders^e^	8 (7)	11 (11)
Health details		
Duration (years) neuro disorder (*M*[s.d.])	11.38 (11.07)	11.59 (9.38)
Comorbid chronic condition^f^	56 (51)	53 (51)
Overall health rating		
Very good	7 (6)	6 (6)
Good	32 (29)	26 (25)
Moderate	53 (48)	44 (42)
Poor	18 (16)	24 (23)
Very poor	1 (1)	4 (4)
Speech Disorder (e.g. aphasia)	22 (20)	15 (14)
Cognitive Status (TICS)^g^ (*M*[s.d.])	27.09 (3.50)	27.09 (3.75)
Current Medications		
Neurology medication	94 (85)	82 (79)
Antidepressant	39 (35)	35 (34)
Anxiolytic^h^	8 (7)	12 (12)
Antipsychotic	3 (3)	4 (4)
Treatment history		
Previous mental health intervention	82 (74)	75 (72)
Current access to psychotherapy	36 (33)	35 (34)
Previous cognitive assessment/rehabilitation	34 (31)	37 (36)
Current access to cognitive rehabilitation	8 (7)	10 (10)
Has a regular carer	31 (28)	33 (32)
Current accesses to the NDIS	43 (39)	31 (30)

NDIS, National Disability Insurance Scheme; s.d., Standard Deviation

a Can report multiple options. Current employment could include full time, part-time or casual work.

b In a relationship included people who were married or de factor. Not in a relationship included single, widowed, divorced/separated.

c A total of 179 (83%) participants had their self-reported diagnosis confirmed by their treating physician. For two participants with a self-reported history of an acquired brain injury (one following motor vehicle accident and one following surgery) their physicians noted they were primarily now being managed for dysautonomia and functional neurological disorder, respectively. One participant's physician noted it was currently unclear whether the patient had epilepsy or psychogenic non-epileptic seizures with investigations ongoing. There were no responses for 34(16%) of participants and 1 (1%) participant was excluded due to physician noting they did not have any of the targeted neurological disorders.

d Treatment group includes 20/111 (18%) with a Traumatic Brain Injury (TBI) and/or 14/111 (13%) with a Stroke. Control includes 19/104 (18%) with a TBI and/or 8/104 (8%) with a Stroke.

e Treatment group includes 5 people with a secondary diagnoses of an ABI and 3 with secondary diagnosis of epilepsy. Control group includes 5 with secondary ABI and 6 with secondary epilepsy.

f Chronic condition (e.g. cardiovascular disease, cancer, diabetes, musculoskeletal condition, respiratory disease).

g Due to significant aphasia two participants were unable to complete the TICS but completed the rest of the application via email or assistance from a carer.

h Majority PRN.

Marginal models were used to examine change in outcomes over time (pre-treatment to post-treatment) between the treatment and control group, for both primary and secondary outcomes. The model was fit using Generalised Estimating Equations (GEE) with an unstructured working correlation matrix to reflect the different rates in change over time. A gamma distribution with a log link function was specified to account for skewness in the dependent variables.

The estimated marginal means (EMMs) and their standard error were displayed for time by group. To determine whether post-treatment improvements were maintained over time in the treatment group, within-group time effects were examined at 3-month follow-up. For all analyses, the level of statistical significance was set at alpha 0.05.

Clinical significance was assessed several ways. First, we calculated the average percentage improvement for each group (e.g. pre-treatment mean score – post-treatment mean score/pre-treatment mean score) from pre-treatment to post-treatment and 3-month follow-up, for the primary and secondary outcomes, using the EMMs from the GEE models. Second, consistent with past research (Dear et al., 2022), the proportion of participants achieving a clinical improvement (defined as ≥25%) and large clinical improvement (defined as ≥50%) on the primary outcomes was also calculated and compared between groups (Karin, Dear, Heller, Gandy, & Titov, 2018b). In addition, deterioration (i.e. symptom increase at post-treatment of ≥30% and within the clinical range) were compared between groups. Based on these outcomes, the number needed to treat (NNT) was calculated. Finally, Hedges *g* effect sizes were calculated for the between-group and within-groups effects.

Prespecified subgroup sensitivity analyses were conducted for primary outcomes based on symptom severity by reporting the clinical effects for participants within the clinical and non-clinical ranges separately. Subgroup analyses based on neurological disorder type were also conducted by reporting the clinical effects across the four primary neurological disorder type (MS, ABI, PD, or epilepsy) separately.

## Results

**Fig. 1.**
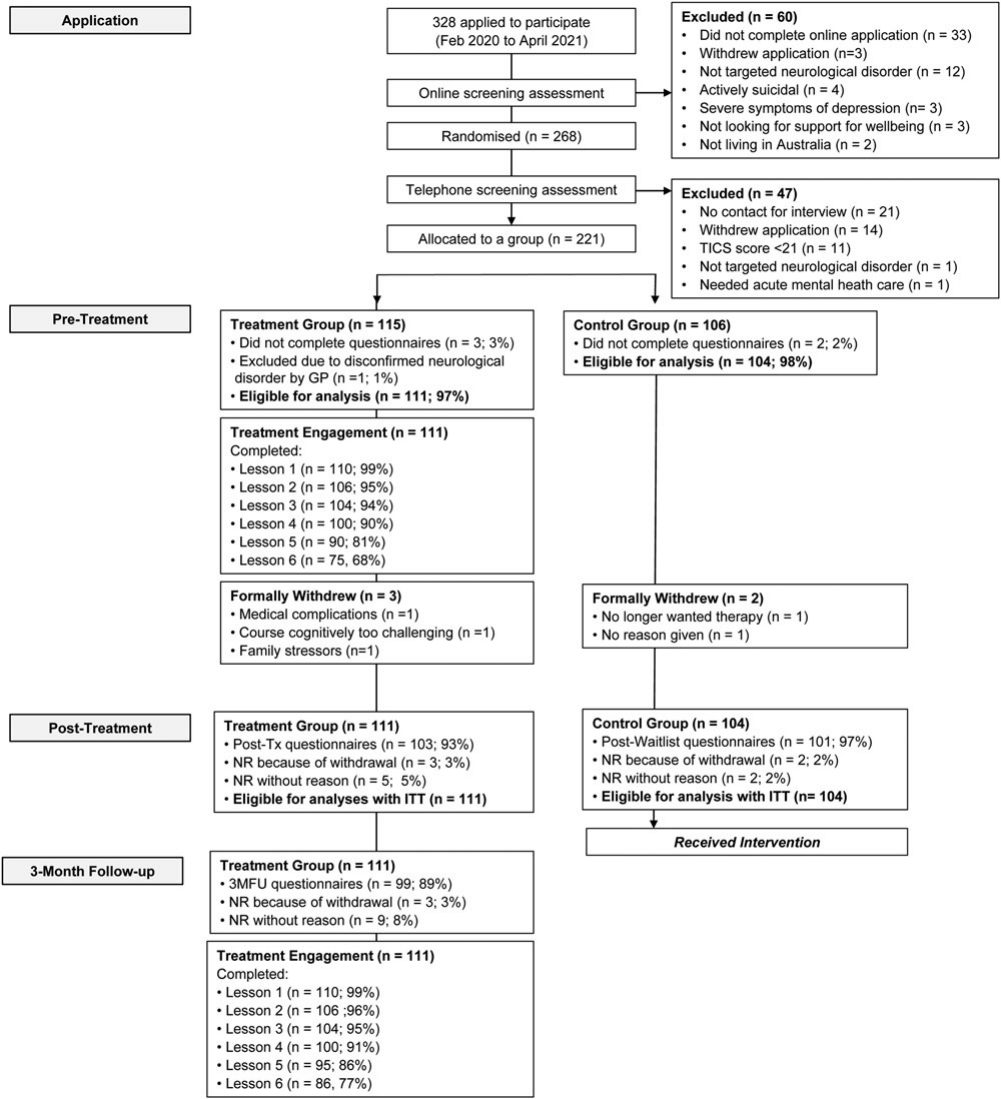
Participant flow from application to 3-month follow-up.

Participants were enrolled from February 2020 to April 2021. Two-hundred and twenty-one participants were randomised to either immediate treatment (*n* = 115) or TAU-WLC (*n* = 106). Of these, 215 participants (treatment *n* = 111; control *n* = 104) completed baseline data (i.e. pre-treatment) and were eligible for analyses (Fig. 1). Adherence, attrition, and treatment satisfaction rates are displayed in (Fig. 1) for the overall sample and by neurological disorder group (online Supplementary Table S2). The mean total clinician contact time per participant in the treatment group was 95.7 min (s.d. = 59.3), which comprised of time answering and making phone calls (*M* = 73.2; s.d. = 60.1; range = 0–258) and time sending or reading secure messages (*M* = 22.42, s.d. = 15.1, range = 0–66).

As displayed in Table 2, the GEE analyses for the primary outcomes revealed significant between-group differences for symptoms of depression (PHQ-9; *p* < 0.001, *g* = 0.62); anxiety (GAD-7; *p* < 0.001, *g* = 0.41) and disability (WHODAS; *p* < 0.001 *g* = 0.31), favouring the treatment group. Participants in the treatment group reported greater symptom improvements on average (24% for depression and anxiety, 15% for disability) compared to controls (−3%, all outcomes). From post-treatment to 3-month follow-up, the treatment group showed no significant time effects, suggesting treatment-related improvements were maintained. As displayed in Table 3 the rates of participants experiencing clinically-significant symptom improvements were significantly higher for treatment participants compared to controls (*p* < 0.01).

**Table 2.** Estimated marginal means, percentage change, and effect sizes with 95% CI for the primary and secondary outcomes

Outcomes		Estimated marginal means (s.e.)							Percentage improvement^a^ (95% CIs)		Between-group Hedge's *g*	Within group Hedge's *g*
Primary	*N*	Pretreatment	Posttreatment	Mean between group diff (95% CI)	*p between*	3MFU	*p Tx p_ost*→*3mfu_*	*p Tx p_re*→*3mfu_*	Pre→Post	Pre→3MFU	Posttreatment	Pre→Post
Depression (PHQ-9)												
Treatment	111	9.02 (0.47)	6.84 (0.43)	3.07 (2.04–4.11)	*p* *<* *0.001*	7.37 (0.56)	*p* *=* *0.102*	*p* *=* *0.007*	24 (15–34)	18 (6–31)	0.62 (0.34–0.90)	0.46 (0.19–0.73)
Control	104	9.64 (0.51)	9.91 (0.53)						−3 (−14 to 8)			0.05 (−0.23 to 0.33)
Anxiety (GAD-7)												
Treatment	111	6.76 (0.47)	5.16 (0.40)	1.87 (0.92–2.81)	*p* *<* *0.001*	4.86 (0.44)	*p* *=* *0.305*	*p* *<* *0.001*	24 (12–35)	28 (15–41)	0.41 (0.13–0.68)	0.35 (0.08–0.62)
Control	104	6.82 (0.49)	7.02 (0.48)						−3 (−17 to 11)			0.04 (−0.24 to 0.32)
Disability (WHODAS)												
Treatment	111	16.91 (0.83)	14.45 (0.87)	3.08 (1.09–5.06)	*p* *<* *0.001*	13.78 (0.85)	*p* *=* *0.163*	*p* *<* *0.001*	15 (4–25)	19 (9–28)	0.31 (0.04–0.59)	0.27 (0.00–0.54)
Control	104	16.97 (0.92)	17.53 (1.01)						−3 (−15 to 8)			0.06 (−0.22 to 0.33)
Secondary^b^												
CogFunction (NeurQoL)												
Treatment	111	23.89 (0.64)	26.39 (0.64)	−2.55 (−3.93 to −1.17)	*p* *<* *0.001*	27.34 (0.72)	*p* *=* *0.228*	*p* *<* *0.001*	−10 (−16 to −5)	−14 (−20 to −8)	0.37 (0.09–0.64)	0.37 (0.10–0.64)
Control	104	23.97 (0.68)	23.83 (0.70)						1 (−5 to 6)			0.02 (−0.26 to 0.30)
Dyscontrol (NeurQol)												
Treatment	111	19.38 (0.58)	17.31 (0.61)	2.95 (1.65–4.25)	*p* *<* *0.001*	17.79 (0.70)	*p* *=* *0.900*	*p* *=* *0.006*	11 (5–17)	8 (1–15)	0.45 (0.17–0.73)	0.33 (0.06–0.60)
Control	104	19.99 (0.64)	20.27 (0.66)						−1 (−8 to 5)			0.04 (−0.24 to 0.32)
PosAffect (NeuroQol)												
Treatment	111	29.36 (0.64)	31.09 (0.68)	−1.74 (−3.24 to −0.24)	*p* *=* *0.13*	30.67 (0.75)	*p* *=* *0.952*	*p* *=* *0.052*	−6 (−10 to −1)	−4 (−10 to 1)	0.24 (−0.04 to 0.51)	0.25 (−0.02 to 0.52)
Control	104	28.76 (0.74)	29.35 (0.76)						−2 (−7 to 3)			0.08 (−0.20 to 0.35)
CogStrategies (CSSQ)												
Treatment	111	33.52 (0.76)	37.15 (0.64)	−2.91 (−4.32 to −1.5)	*p* *=* *0.002*	37.00 (0.69)	*p* *=* *0.772*	*p* *<* *0.001*	−11 (−15 to −7)	−10 (−14 to −6)	0.41 (0.14–0.69)	0.49 (0.21–0.76)
Control	104	33.25 (0.82)	34.24 (0.72)						−3 (−7 to 1)			0.13 (−0.15 to 0.40)

3MFU, 3 month follow-up; CogFunction, Cognitive Function; CI, Confidence Intervals; CCSQ, Compensatory Cognitive Strategies Questionnaire; Dyscontrol, Emotional and Behavioural Dyscontrol; GAD-7, Generalised Anxiety Disorder Scale 7-Item; Mean between group diff, Mean group difference between treatment and control group; Neuro-QoL, Quality of Life in Neurological Disorders; PHQ-9, Patient Health Questionnaire 9-Item; Post, Posttreatment; PostAffect, Positive Affect & Wellbeing; Pre, Pre-treatment; Tx, Treatment; s.e., Standard Error; WHODAS, World Health Organisation Disability Assessment Schedule 2.0

a Percentage improvement is the average percentage change in mean scores (e.g. pre-treatment – post-treatment/ pre-treatment).

b Three of the secondary measures (Cognitive Function -NeuroQoL, Positive Affect and Wellbeing –NeuroQoL and the Compensatory Cognitive Strategies Questionnaire) are scored such that higher scores are indicative of improvements in these domains. Whereas, the Emotional and Behavioural Dyscontrol –NeuroQo is scored such that higher scores are indicative of higher levels of difficulties in this area.

**Table 3.** Clinical improvements, deteriorations and NNT with 95% CIs for the primary outcomes

	≥25% improvement				≥50% improvement				≥ 30% deterioration	
	Posttreatment (95% CI)	*p between*	3MFU (95% CI)	NNT (95% CI)	Posttreatment (95% CI)	*p Tx post*→*3mfu*	3MFU (95% CI)	NNT	Posttreatment (95% CI)	*p Between*
Depression (PHQ-9)										
Treatment	48.65 (39.04–58.25)	*p* *=* *0.001*	48.83 (38.87–58.79)	4.13 (2.68–8.99)	32.43 (23.62–41.25)	*p* *<* *0.001*	25.05 (16.21–33.88)	3.95 (2.79–6.73)	5.05 (0.59–9.5)	*p* *=* *0.151*
Control	24.42 (15.96–32.89)				7.12 (1.82–12.41)				10.77 (4.74–16.8)	
Anxiety (GAD-7)										
Treatment	48.83 (39.39–58.27)	*p* *=* *0.005*	56.76 (47.44–66.07)	5.2 (3.11–15.86)	36.04 (26.89–45.18)	*p* *<* *0.001*	42.52 (33.28–51.76)	4.47 (2.98–8.96)	5.41 (1.2–9.61)	*p* *=* *0.385*
Control	29.62 (20.67–38.56)				13.65 (6.99–20.32)				8.46 (3.03–13.89)	
Disability (WHODAS)										
Treatment	40.90 (31.69–50.11)	*p* *<* *0.001*	38.92 (28.84–49.00)	4.2 (2.8–8.41)	17.48 (10.33–24.62)	*p* *=* *0.006*	16.76 (9.45–24.06)	7.89 (4.78–22.59)	10.99 (5.11–16.87)	*p* *=* *0.646*
Control	17.12 (9.67–24.56)				4.81 (0.70–8.92)				13.08 (6.5–19.66)	

%, percentage; 3MFU, 3 month follow-up; CI, Confidence Intervals; GAD-7, Generalised Anxiety Disorder Scale 7-Item; NNT, number needed to treat; PHQ-9, Patient Health Questionnaire 9-Item; Post, Posttreatment; Pre, Pre-treatment; Tx, Treatment; WHODAS, World Health Organisation Disability Assessment Schedule 2

In relation to clinical severity relevance, subgroup analyses found significant between-group differences, for participants within both the clinical and non-clinical baseline ranges on the primary outcomes (*p* < 0.05; Table 4). For depression, anxiety, and disability the clinical effects were larger for participants within the clinically relevant range at baseline (between groups Hedges *g* = 0.82, *g* = 0.75, *g* = 0.51, respectively) compared to the non-clinical range (*g* = 0.45, *g* = 0.29, *g* = 0.28).

**Table 4.** Subgroup severity analyses for primary outcomes

Severity subgroup		Estimated marginal means (s.e.)							Percentage improvement^a^ (95% CIs)		Between-group Hedge's *g*	Within group Hedge's *g*
Depression (PHQ-9)		Pretreatment	Posttreatment	Mean between group diff (95% CI)	*p between*	3MFU	*p Tx p_ost*→*3mfu_*	*p Tx p_re*→*3mfu_*	Pre→Post	Pre→3MFU	Posttreatment	Pre→Post
>9 clinical												
Treatment	44	14.07 (0.53)	9.62 (0.77)	3.83 (2.61–5.05)	*p* *<* *0.001*	9.91 (1.01)	*p* *=* *0.562*	*p* *=* *0.001*	32% (21–42)	30% (15–44)	0.82 (0.39–1.24)	1.03 (0.57–1.48)
Control	51	14.04 (0.45)	13.45 (0.62)						4% (−5 to 13)			0.15 (−0.25 to 0.55)
≤9 non-clinical												
Treatment	67	5.70 (0.26)	5.01 (0.37)	1.5 (0.5–2.49)	*p* *=* *0.003*	5.7 (0.5)	*p* *=* *0.068*	*p* *=* *0.988*	12% (−1 to 25)	0% (−17 to 18)	0.45 (0.07–0.83)	0.27 (−0.08 to 0.61)
Control	53	5.40 (0.36)	6.51 (0.51)						−21% (−39 to −2)			0.34 (−0.05 to 0.74)
Anxiety (GAD-7)												
>9 clinical												
Treatment	29	13.62 (0.59)	9.29 (0.85)	2.86 (1.75–3.97)	*p* *=* *0.002*	9.07 (1)	*p* *=* *0.721*	*p* *<* *0.001*	32% (20–44)	33% (19–48)	0.75 (0.23–1.28)	1.13 (0.90–1.36)
Control	33	13.06 (0.46)	12.15 (0.57)						7% (−2 to 15)			0.30 (−0.20 to 0.79)
≤9 clinical												
Treatment	82	4.33 (0.28)	3.69 (0.34)	0.94 (0.12–1.77)	*p* *=* *0.006*	3.37 (0.35)	*p* *=* *0.307*	*p* *=* *0.572*	15% (−1 to 30)	22% (6–38)	0.29 (−0.04 to 0.61)	0.23 (−0.08 to 0.54)
Control	71	3.92 (0.31)	4.64 (0.42)						−18% (−40 to 3)			0.22 (−0.11 to 0.56)
Disability (WHODAS)												
>9												
Treatment	87	19.85 (0.81)	17.02 (0.92)	4.32 (2.35–6.29)	*p* *<* *0.001*	16.02 (0.91)	*p* *=* *0.088*	*p* *<* *0.001*	14% (5–23)	19% (10–28)	0.51 (0.19–0.82)	0.36 (0.06–0.67)
Control	78	20.72 (0.87)	21.34 (1.01)						−3% (−13 to 7)			0.08 (−0.24 to 0.40)
≤9												
Treatment	24	6.25 (0.50)	5.13 (0.57)	0.95 (−0.53 to 2.43)	*p* *=* *0.041*	5.68 (1)	*p* *=* *0.526*	*p* *=* *0.49*	18% (0–36)	9% (−22 to 41)	0.28 (−0.29 to 0.84)	0.42 (−0.17 to 1.00)
Control	26	5.73 (0.51)	6.08 (0.76)						−6% (−32 to 20)			0.1 (−0.45 to 0.66)

3MFU, 3 month follow-up; CI, Confidence Intervals; GAD-7, Generalised Anxiety Disorder Scale 7-Item; PHQ-9, Patient Health Questionnaire 9-Item; Post, Posttreatment; Pre, Pre-treatment; Tx, Treatment; s.e., Standard Error; WHODAS, World Health Organisation Disability Assessment Schedule 2.0

a Percentage improvement is the average percentage change in mean scores (e.g. pre-treatment – post-treatment/pre-treatment).

>9 is considered the clinical cut-off range for the primary outcome measures. ≤9 is considered the non-clinical range.

Subgroup analyses revealed evidence of significant group differences on the primary outcomes across the neurological disorders, with a few exceptions (online Supplementary Table S3). For ABI, all pattern of results remained consistent with the overall trends. For MS participants post-treatment results remained consistent but from post-treatment to 3-month follow-up effects for depression significantly reduced (*p* = 0.007). For people with PD there were no significant group-differences on the primary outcomes. For epilepsy, there were significant group-differences for depression (*p* = 0.006) and anxiety (*p* = 0.07) but not for disability (*p* = 0.112). However significant improvements in disability from pre-treatment to 3-month follow-up emerged (*p* = 0.019).

Across the primary outcomes, there were no significant group-differences between the treatment and control groups in the deterioration rates, which were low (Table 3). On post-treatment questionnaires five treatment participants (5%) self-reported an increase in their symptoms related to taking part in the course, with four noting this was part of the therapeutic process, and one noting that external factors had impacted their mood.

As displayed in Table 2, the GEE analysis revealed significant between-group differences in secondary outcomes of cognitive function (*p* < 0.001, *g* = 0.37); emotional and behavioural dyscontrol (*p* < 0.001, *g* = 0.45) and cognitive strategy use (*p* = 0.002, *g* = 0.41), all favouring treatment. On average, participants in the treatment group also reported greater pre-treatment to post-treatment symptom improvements on these measures (≥10%) compared to controls (≥3%). Treatment-related improvements were maintained at 3-month follow-up. There were no significant between-group differences for the measure of positive affect and wellbeing (*p* = 0.13).

## Discussion

The results of this RCT provide preliminary support for the acceptability and efficacy of a novel transdiagnostic and internet-delivered psychological intervention for adults with neurological disorders. The treatment group reported significant improvements in primary outcomes (between group Hedges *g*: depression = 0.62; anxiety = 0.41; disability = 0.31) compared to those in the control group, with improvements maintained at 3-month follow-up. Regarding secondary outcomes, the treatment group reported greater improvements on measures of cognitive function, emotional and behavioural dyscontrol, and the use of compensatory cognitive strategies, compared to controls. However, there was no difference in positive affect and wellbeing. The results suggest the intervention was highly acceptable, with high rates of lesson completion and satisfaction with the intervention.

The results from this Phase II clinical trial extend on a previous Phase I examination of this intervention using an open trial design, where significant within-group improvements were observed (Gandy et al., 2020). The use of a control group in the current study indicates that the observed symptom improvements were not simply due to time or processes of natural remission. Of note, about half the sample (48%) experienced a clinical improvement (defined as ≥25% improvement) in symptoms of depression and anxiety, with over one third of participants experiencing a large clinical improvement (defined as ≥50% improvement). These findings compare well with meta-analytic pooled estimates of between 37% to 48% of participants making a large clinical improvement (≥50%) in depressive symptoms following internet-delivered CBT programmes for depression in the general population (Karyotaki et al., 2021).

The results of this trial highlight the value of a transdiagnostic approach. Given that no lower threshold for symptom severity was imposed, some participants entered the trial without clinically significant symptoms on one or more domains (e.g. clinically significant anxiety symptoms, but not depressive symptoms). Despite our main analyses including individuals in the non-clinical range, significant small to moderate treatment effects were observed. There are currently no agreed benchmarks for CBT for neurological disorders. Importantly, however, the size of these effects are consistent with recent meta-analytic small to medium pooled effect size estimates of CBT for depression and anxiety in people with PD (Ghielen et al., 2019; Zhang, Yang, Song, & Jin, 2020), MS (Ghielen et al., 2019) and post-stroke (Wang et al., 2018), as well as disability for patients with chronic pain (Gandy et al., 2022). Moreover, our sensitivity analyses revealed larger treatment effects when isolated to participants whose baseline scores were in the clinical range, with large effects observed for changes in depression (*g* = 0.82) and moderate effects for anxiety (*g* = 0.75) and disability (*g* = 0.51).

Overall, our observed clinical effects on the primary outcomes are also largely consistent with disorder-specific RCTs of iCBT programmes in neurological disorders for depression in people with MS and epilepsy (Fischer et al., 2015; Meyer et al., 2019; Schröder et al., 2014) and functional disability in PD (Kraepelien et al., 2020). However, this is RCT presents the novel findings of clinically significant effects following a transdiagnostic intervention. The broad acceptability and efficacy of the current intervention also supports the transdiagnostic approach to care, whereby a single intervention can be suitable for patients with a variety of neurological disorders and comorbidities and simultaneously targets multiple mental health and functional domains. This has the potential to reduce the need for developing and testing multiple disorder- and domain-specific interventions, which from a public health perspective have more limited scalability and scope.

It is noteworthy that significant treatment-related improvements were also observed in three of the four secondary outcomes of cognitive function (*g* = 0.37), emotional and behavioural dyscontrol (*g* = 0.45), and use of cognitive strategies (*g* = 0.41). These findings are important, given mixed evidence that anti-depressant medications can improve cognitive or functional outcomes for patients with neurological disorders (Price et al., 2011) and often come with unwanted side effects. However, the current findings require replication and caution is needed before firm conclusions are drawn in this area. Furthermore, no differences between the treatment and control groups were observed for the measure of positive affect and wellbeing. These findings may suggest the impact of intervention on mental health and functional outcomes may not extend to the broader domain of life satisfaction.

The findings of the subgroup analyses are also encouraging, with evidence of consistent acceptability across the neurological disorder subgroups and broad evidence of efficacy across the primary outcomes, with a few notable exceptions. Namely, participants with PD did not experience significant improvements across the primary outcomes. However, compared to the other subgroups these participants reported lower levels of symptoms at baseline, which may present less opportunity for change. The lower baseline severity of the PD subgroup may also be related to sample bias. Reflecting this, recruitment occurred primarily via online promotion and only about 10% of participants were aged over 70 years of age. Thus, our sample may not represent older and/more progressed participants with PD within the community. Despite this, the rates of intervention satisfaction and lesson completion were still high in people with PD within the current study. For instance, >95% of people with PD noted the course was worth their time and >70% completed all 6 lessons at post-treatment, which suggest these participants found the intervention acceptable and engaging. Importantly, all subgroup analyses should be interpreted as preliminary trends only given the relatively small sample sizes and subsequently underpowered analyses. Future studies with larger sample sizes of individual neurological disorder groups are needed before firm conclusions can be drawn in this area. In addition, moderator analyses are needed to identify characteristics (e.g. sex, age, disorder type, health status, symptom severity) of intervention non-responders, who may require alternative care options.

The findings of this trial should be considered in the context of several limitations. The present study relied on standardised self-report outcome measures. Future research may benefit from more objective measures, such as the use of formal diagnostic assessments of mental health disorders and cognitive function. However, the use of self-report measures of psychological difficulties and symptom severity is accepted practice in these types of clinical trials (Mehta et al., 2019), are an important predictor of health service use (Andelic, Soberg, Berntsen, Sigurdardottir, & Roe, 2014), and reduces the burden of complex assessment for participants and service providers. The current study only investigated the intervention in four common neurological disorder groups and excluded participants with very severe depression or severe cognitive impairment. Participants were also informed that the intervention was based on self-management principles, and on average participants reported their overall health within the moderate range. Thus, the findings may not generalise to neurological disorders more broadly, people with perceived poorer health status, or people not interested in self-management. Future trials with more inclusive study criteria (e.g. other neurological disorders, functional neurological disorders) and more heterogenous and varied samples (e.g. self and doctor referred patients, more severe patients) will be an important next step. The study also utilised a relatively short follow-up period of 3 months. Future trials with longer follow-up periods are necessary to assess whether improvements are maintained in the long term. Consistent with previous research in this area, we utilised a treatment-as-usual waitlist control group, which means we cannot be certain to what extent the intervention contributed to improvements versus other more general factors, such as therapist attention and expectancy. Thus, future clinical trials with active control groups (e.g. a supportive counselling arm) are needed before firm conclusions can be reached about the specific efficacy of the current intervention. Finally, although the overall study was adequately powered, subgroup analyses were underpowered, which precludes firm conclusions about the specificity of outcomes by symptom severity and neurological disorder group, and the observations reported are trends only.

Notwithstanding these limitations, the current study provides preliminary support for the efficacy of a transdiagnostic and internet-delivered psychological intervention to address depression, anxiety, and disability among people with neurological disorders. Importantly, participants found the intervention to be highly satisfying and engaging. These results were achieved with only a modest amount of clinician time per patient, indicating the public health potential of this model of care. These findings point to the potential value of such interventions as a referral option for neurologists and neurorehabilitation clinics treating many patients with mental health difficulties, but who often struggle to access appropriate psychological care for these patients (Chan et al., 2022; Gandy et al., 2020). This is particularly pertinent given the increased recognition for the need for greater supportive care options for neurology patients (WHO, 2020) and emerging disciplines like neuropalliative care (Creutzfeldt et al., 2018). Future research examining the potential of internet-delivered psychological interventions within more diverse samples of people with neurological disorders, utilising clinical trials with active controls, and trials within routine neurology care settings is warranted.

## Supplementary material

For supplementary material accompanying this paper visit https://doi.org/10.1017/S0033291723000338. click here to view supplementary material
