# Supplementary material for: The wellbeing neuro course: a randomised controlled trial of an internet-delivered transdiagnostic psychological intervention for adults with neurological disorders
Source: Psychol Med. 2023 Mar 1;53(14):6817–27. doi: 10.1017/S0033291723000338 (PMC10600819; doi:10.1017/S0033291723000338)
Supplement: Supplementary file 1 [file S0033291723000338sup001.docx]

**Table S 1.** Timetable and Content of the Wellbeing Neuro Course

**Method S 1.** Copy of the Compensatory Cognitive Strategies Questionnaire

**Table S 2**: Lesson completion and satisfaction ratings by neurological disorder group

**Table S 3.** Subgroup neurological disorder analyses by neurological disorder group for the primary outcomes.

**Table S 4:** Estimated marginal means, percentage change, and effect sizes with 95% CI for the neurological specific measures for validation purposes

**Table S 5.** Subgroup neurological disorder analyses for the neurological-disorder measures.

Supplementary

Table 1. Timetable and Content of the Wellbeing Neuro Course

| **Lesson** | **Time Before Next Lesson** | **Lesson Content** | **Primary Skill Taught** | **Additional Resources** |
| --- | --- | --- | --- | --- |
| **1** | 1 week | Introduction to self-management. Education about how neurological disorders can impact emotional and cognitive wellbeing. Introduction of a CBT model and explanation of the functional relationship between thought, physical, behavioural and neurological symptoms. Case examples and tips to assist identifying their own symptoms and how their symptoms interact. | - Symptom identification  - Symptom formulation | -Managing Sleep  -Managing Wellbeing – COVID-19 |
| **2** | 2 weeks | Introduction to structured problem solving as a means of managing the impact of neurological symptoms. Instructions and examples for using structured problem solving in everyday life. Introduction to compensatory cognitive rehabilitation skills for managing common cognitive problems e.g., forgetting. | - Structured Problem Solving | -Managing Cognitive Problems |
| **3** | 2 weeks | Introduction to the basic principles of cognitive therapy and importance of managing thoughts to manage one’s wellbeing and impact of neurological disorder. Instructions and case examples for monitoring and challenging thoughts. | - Thought monitoring  - Thought challenging | - |
| **4** | 2 weeks | Introduction to the physical symptoms of depression (i.e., hypo-arousal) and anxiety/anger (i.e.hyper-arousal) and relationship with wellbeing and managing neurological symptoms. Instructions about managing physical symptoms via scheduling pleasant, physical and cognitive activities. Instructions for using de-arousal strategies such as controlled breathing. | - Activity scheduling  - Controlled relaxation | -Assertive Communication |
| **5** | 2 weeks | Introduction to the behavioural symptoms of anxiety, depression and neurological disorders. Explanation of the overdoing-underdoing cycle of activity levels and issues around the fear and the avoidance of social, physical and cognitive activities. Instructions for activity pacing and gradually tackling avoidance. | - Activity pacing | -Managing Avoidance |
| **6** | 1 week till post-treatment | Information about the occurrence of setbacks in thought, physical, behavioural and neurological symptoms. Information about the signs of setbacks and instructions for creating setback plans. Information about the importance of continued structured problem solving and revisions/practice of skills into the future. | - Structured Problem Solving  - Relapse prevention | -Managing Anger |

Supplementary

Method 1.Copy of the Compensatory Cognitive Strategies Questionnaire

| CCSQ  (eCentreClinic) | | |
| --- | --- | --- |
| Over the past week, how often did you use the following strategies to help solve cognitive problems, such as problems forgetting to do things? | | |
| *The rating scale is as follows:*  1 = Never  2 = Rarely  3 = Sometimes  4 = Often  5 = Always  In the past week … | | |
| 1 | Planned my day | 1 2 3 4 5 |
| 2 | Stuck to a routine | 1 2 3 4 5 |
| 3 | Used reminders or alarms. | 1 2 3 4 5 |
| 4 | Used cues (e.g., post-it notes) | 1 2 3 4 5 |
| 5 | Repeated information I needed to remember. | 1 2 3 4 5 |
| 6 | Broke down information or tasks into smaller parts. | 1 2 3 4 5 |
| 7 | Limited distractions around me. | 1 2 3 4 5 |
| 8 | Recorded information I needed to remember (e.g., took photos). | 1 2 3 4 5 |
| 9 | Kept important personal items (e.g., keys) in the same place as not to lose them | 1 2 3 4 5 |
| 10 | Asked other people for help when I needed it. | 1 2 3 4 5 |

Supplementary

Table 2: Lesson completion and satisfaction ratings by neurological disorder group

|  | Post-treatment | | | | 3 Month follow-up | | | |
| --- | --- | --- | --- | --- | --- | --- | --- | --- |
| Completed | MS | ABI | PD | Epilepsy | MS | ABI | PD | Epilepsy |
|  | n =41 | n =29 | n =25 | n =16 | n =41 | n =29 | n =25 | n =16 |
|  | Post-treatment n (%) | | | | 3-month follow-up n (%) | | | |
| Lesson 1 | 40 (98%) | 29 (100%) | 25(100%) | 16 (100%) | 40 (98%) | 29 (100%) | 25(100%) | 16 (100%) |
| Lesson 2 | 39 (95%) | 28 (87%) | 25 (100%) | 14 (88%) | 39 (95%) | 28 (87%) | 25 (100%) | 14 (88%) |
| Lesson 3 | 39 (95%) | 27 (93%) | 24 (96%) | 14 (88%) | 39 (95%) | 27 (93%) | 24 (96%) | 14 (88%) |
| Lesson 4 | 36 (88%) | 27 (93%) | 24 (96%) | 13 (81%) | 36 (88%) | 27 (93%) | 24 (96%) | 13 (81%) |
| Lesson 5 | 33 (80%) | 24 (83%) | 21 (84%) | 12 (75%) | 35 (85%) | 25 (86%) | 22 (88%) | 13 (81%) |
| Lesson 6 | 29 (71%) | 20 (69%) | 18 (72%) | 8 (50%) | 34 (83%) | 21 (72%) | 21 (84%) | 10 (63%) |
| Course Satisfaction* |  |  |  |  |  |  |  |  |
| Very satisfied | 24/37 (65%) | 17/27 (63%) | 17/23 (74%) | 7/15 (47%) | NA | NA | NA | NA |
| Satisfied | 10/37 (27%) | 8/27 (30%) | 6/23 (26%) | 7/15 (47%) | NA | NA | NA | NA |
| Neutral | 3/37 (8%) | 2/27 (7%) | 0/23 (0%) | 1/15 (7%) | NA | NA | NA | NA |
| Dissatisfied | 0/37 (0%) | 0/27 (0%) | 0/23 (0%) | 0/15 (0%) | NA | NA | NA | NA |
| Very dissatisfied | 0/37 (0%) | 0/27 (0%) | 0/23 (0%) | 0/15 (0%) | NA | NA | NA | NA |
| Worth Time | 34/37 (92%) | 25/27 (93%) | 22/23 (96%) | 15/15 (100%) | NA | NA | NA | NA |
| Recommend | 36/37 (97%) | 27/27 (100%) | 23/23 (100%) | 15/15 (100%) | NA | NA | NA | NA |

ABI= Acquired Brian Injury; MS= Multiple Sclerosis; NA= Not Available. PD = Parkinson’s Disease.

*Based on treatment participants providing feedback.

Supplementary

Table 3. Sub-group neurological disorder analyses by neurological disorder group for the primary outcomes.

|  | | Estimated Marginal Means (SE) | | | | |  |  | Percentage improvement  (95% CIs)* | |
| --- | --- | --- | --- | --- | --- | --- | --- | --- | --- | --- |
|  |  |  |  |  |  |  |  |  |  |  |
| **Primary Outcomes** | N | Pretreatment | Posttreatment | Mean between group diff (95% CI) | *P between* | 3MFU | *P Tx p_ost→3mfu_* | *P Tx p_re→3mfu_* | Pre→Post | Pre→3MFU |
| **Depression**  **(PHQ-9)** |  |  |  |  |  |  |  |  |  |  |
| **MS** |  |  |  |  |  |  |  |  |  |  |
| Treatment | 41 | 9.37 (0.75) | 7.65 (0.78) | 2.29 (0.8, 3.78) | *p=.006* | 9.05 (0.96) | *p=.007* | *p=.696* | 18% (2, 35) | 3% (-17, 24) |
| Control | 44 | 9.34 (0.61) | 9.94 (0.76) |  |  |  |  |  | -6% (-22, 9) | -- |
| **ABI** |  |  |  |  |  |  |  |  |  |  |
| Treatment | 29 | 10.45 (0.93) | 7.93 (0.88) | 3.74 (1.46, 6.02) | *p=.001* | 7.86 (1.13) | *p=.809* | *p=.04* | 24% (8, 41) | 25% (3, 46) |
| Control | 24 | 11.92 (1.03) | 11.67 (1.16) |  |  |  |  |  | 2% (-17, 21) | -- |
| **PD** |  |  |  |  |  |  |  |  |  |  |
| Treatment | 25 | 6.44 (0.76) | 5.12 (0.68) | 1.27 (-0.57, 3.11) | *p=.171* | 6.05 (0.81) | *p=.188* | *p=.653* | 20% (0, 41) | 6% (-19, 31) |
| Control | 18 | 6.39 (1.27) | 6.39 (0.94) |  |  |  |  |  | 0% (-29, 29) | -- |
| **Epilepsy** |  |  |  |  |  |  |  |  |  |  |
| Treatment | 16 | 9.56 (1.35) | 5.46 (0.98) | 5.56 (3, 8.12) | *p=.006* | 4.22 (0.74) | *p=.256* | *p<.001* | 43% (23, 63) | 56% (41, 71) |
| Control | 18 | 10.56 (1.39) | 11.02 (1.30) |  |  |  |  |  | -4% (-29, 20) | -- |
| **Anxiety (GAD-7)** |  |  |  |  |  |  |  |  |  |  |
| **MS** |  |  |  |  |  |  |  |  |  |  |
| Treatment | 41 | 6.78 (0.77) | 5.24 (0.70) | 1.1 (-0.25, 2.45) | *p=.032* | 5.84 (0.80) | *p=.263* | *p=.16* | 23% (2, 43) | 14% (-9, 37) |
| Control | 44 | 6.09 (0.66) | 6.34 (0.69) |  |  |  |  |  | -4% (-26, 18) | -- |
| **ABI** |  |  |  |  |  |  |  |  |  |  |
| Treatment | 29 | 7.66 (0.89) | 5.84 (0.88) | 3.28 (0.95, 5.61) | *p=.018* | 5.18 (0.99) | *p=.228* | *p=.011* | 24% (1, 46) | 32% (7, 58) |
| Control | 24 | 8.79 (1.14) | 9.13 (1.19) |  |  |  |  |  | -4% (-30, 23) | -- |
| **PD** |  |  |  |  |  |  |  |  |  |  |
| Treatment | 25 | 5.64 (0.98) | 4.65 (0.74) | 0.13 (-1.48, 1.74) | *p=.179* | 3.85 (0.64) | *p=.178* | *p=.015* | 18% (-8, 43) | 32% (9, 54) |
| Control | 18 | 4.72 (0.94) | 4.78 (0.82) |  |  |  |  |  | -1% (-35, 33) | -- |
| **Epilepsy** |  |  |  |  |  |  |  |  |  |  |
| Treatment | 16 | 6.81 (1.21) | 4.49 (0.82) | 3.64 (1.62, 5.66) | *p=.07* | 3.35 (0.85) | *p=.148* | *p=.003* | 34% (10, 58) | 51% (26, 75) |
| Control | 18 | 8.06 (1.27) | 8.13 (1.03) |  |  |  |  |  | -1% (-26, 24) | -- |
| **Disability (WHODAS)** |  |  |  |  |  |  |  |  |  |  |
| **MS** |  |  |  |  |  |  |  |  |  |  |
| Treatment | 41 | 18.39 (1.46) | 15.97 (1.43) | 2.65 (-0.55, 5.84) | *p=.005* | 15.57 (1.36) | *p=.598* | *p<.001* | 13% (-2, 28) | 15% (1, 30) |
| Control | 44 | 18.25 (1.42) | 18.62 (1.63) |  |  |  |  |  | -2% (-20, 16) | -- |
| **ABI** |  |  |  |  |  |  |  |  |  |  |
| Treatment | 29 | 20.14 (1.32) | 17.42 (1.5) | 3.12 (-1.09, 7.32) | *p=.002* | 15.39 (1.68) | *p=.137* | *p=.005* | 13% (-1, 28) | 24% (7, 40) |
| Control | 24 | 19.00 (2.04) | 20.54 (2.14) |  |  |  |  |  | -8% (-30, 14) | -- |
| **PD** |  |  |  |  |  |  |  |  |  |  |
| Treatment | 25 | 12.48 (1.25) | 10.14 (1.44) | 1.75 (-1.76, 5.26) | *p=.312* | 11.55 (1.56) | *p=.189* | *p=.381* | 19% (-4, 41) | 7% (-17, 32) |
| Control | 18 | 12.78 (1.97) | 11.89 (1.79) |  |  |  |  |  | 7% (-21, 34) | -- |
| **Epilepsy** |  |  |  |  |  |  |  |  |  |  |
| Treatment | 16 | 14.19 (2.47) | 11.90 (2.67) | 4.58 (0.41, 8.74) | *p=.112* | 9.78 (2.23) | *p=.091* | *p=.019* | 16% (-21, 53) | 31% (0, 62) |
| Control | 18 | 15.33 (1.79) | 16.48 (2.12) |  |  |  |  |  | -7% (-35, 20) | -- |

* Percentage improvement is the average percentage change in mean scores (e.g., pre-treatment – post-treatment/ pre-treatment).

3MFU = 3 month follow-up; CI = Confidence Intervals; GAD-7 = Generalized Anxiety Disorder Scale 7-Item; PHQ-9 = Patient Health Questionnaire 9-Item; Post = Posttreatment;

ABI = Acquired Brain Injury; MS = Multiple Sclerosis, PD = Parkinson’s Disease; Pre= Pre-treatment; Tx = Treatment; SE = Standard Error; WHODAS = World Health Organisation Disability Assessment Schedule 2.0.

Supplementary

Table 4: Estimated marginal means, percentage change, and effect sizes with 95% CI for the neurological specific measures for validation purposes

| Outcomes | | Estimated Marginal Means (SE) | | | | |  |  | Percentage improvement* (95% CIs) | |  | Within group Hedge's g | |
| --- | --- | --- | --- | --- | --- | --- | --- | --- | --- | --- | --- | --- | --- |
|  |  |  |  |  |  |  |  |  |  |  |  |  |  |
|  | N | Pretreatment | Posttreatment | Mean between group diff (95% CI) | P between | 3MFU | P Tx  p_ost→3mfu_ | P Tx  p_re→3mfu_ | Pre→Post | Pre→3MFU | Posttreatment | Pre→Post | Pre→3MFU |
| **Depression**  **(NDDI-E)** |  |  |  |  |  |  |  |  |  |  |  |  |  |
| Treatment | 111 | 14.6 (0.33) | 13.07 (0.38) | 1.71 (0.88,2.53) | p=.002 | 12.94 (0.38) | p=.836 | p<.001 | 10 (5,16) | 11 (6,16) | 0.42 (0.14,0.69) | 0.41 (0.14,0.68) | 0.45 (0.18,0.72) |
| Control | 104 | 15.32 (0.42) | 14.78 (0.42) |  |  | .. |  |  | 4 (-2,9) | .. |  | 0.13  (-0.15,0.40) |  |
| **Anxiety (BrEASI)** |  |  |  |  |  |  |  |  |  |  |  |  |  |
| Treatment | 111 | 7.10 (0.49) | 5.27 (0.44) | 2.28 (1.26,3.29) | p<.001 | 5.61 (0.49) | p=.341 | p=.001 | 26 (14,38) | 21 (8,34) | 0.46 (0.18,0.74) | 0.37 (0.10,0.64) | 0.30 (0.03,0.57) |
| Control | 104 | 7.13 (0.53) | 7.55 (0.52) |  |  | .. |  |  | -6 (-20,8) | .. |  | 0.08 (-0.20,0.36) |  |

* Percentage improvement is the average percentage change in mean scores (e.g., pre-treatment – post-treatment/ pre-treatment).

3MFU = 3 month follow-up; brEASI = The Brief Epilepsy Anxiety-modified; CI = Confidence Intervals; NDDI-E= Neurological Depressive Disorders Inventory-Epilepsy; Post = Posttreatment; Pre= Pre-treatment; Tx = Treatment; SE = Standard Error.

**^a^** The brEASI underwent one slight medication where the word “seizure” was replaced with “neurological symptoms” on two occasions to make the scale appropriate to all neurological disorders

Supplementary

Table 5.Subgroup neurological disorder analyses for the neurological-disorder measures.

|  | | Estimated Marginal Means (SE) | | | | |  |  | Percentage improvement * (95% CIs) | |
| --- | --- | --- | --- | --- | --- | --- | --- | --- | --- | --- |
|  |  |  |  |  |  |  |  |  |  |  |
| **Depression**  **(NDDI-E)** | N | Pretreatment | Posttreatment | Mean between group diff (95% CI) | P between | 3MFU | P Tx  p_ost→3mfu_ | P Tx  p_re→3mfu_ | Pre→Post | Pre→3MFU |
| **MS** |  |  |  |  |  |  |  |  |  |  |
| Treatment | 41 | 14.71 (0.55) | 12.92 (0.62) | 2.28 (0.56, 3.99) | p=.110 | 14.09 (0.66) | p=.006 | p=.148 | 12% (4, 20) | 4% (-5, 13) |
| Control | 44 | 15.93 (0.6) | 14.75 (0.62) |  |  |  |  |  | 7% (0, 15) | .. |
| **ABI** |  |  |  |  |  |  |  |  |  |  |
| Treatment | 29 | 16.07 (0.63) | 14.56 (0.73) | 1.83 (0.62, 3.04) | p=.008 | 13.28 (0.70) | p=.07 | p<.001 | 9% (1, 18) | 17% (9, 26) |
| Control | 24 | 16.00 (0.85) | 16.67 (0.76) |  |  |  |  |  | -4% (-14, 5) | .. |
| **PD** |  |  |  |  |  |  |  |  |  |  |
| Treatment | 25 | 13.20 (0.62) | 11.61 (0.74) | 0.17 (-1.84, 2.18) | p=.220 | 12.17 (0.69) | p=.306 | p=.092 | 12% (1, 23) | 8% (-2, 18) |
| Control | 18 | 12.50 (0.99) | 11.78 (1.03) |  |  |  |  |  | 6% (-10, 22) | .. |
| **Epilepsy** |  |  |  |  |  |  |  |  |  |  |
| Treatment | 16 | 13.81 (0.69) | 13.04 (0.90) | 2.11 (0.61, 3.61) | p=.548 | 10.63 (0.77) | p=.058 | p=.002 | 6% (-7, 18) | 23% (12, 34) |
| Control | 18 | 15.72 (1.00) | 15.32 (0.88) |  |  |  |  |  | 3% (-8, 14) | .. |
| **Anxiety**  **(BrEASI)** |  |  |  |  |  |  |  |  |  |  |
| **MS** |  |  |  |  |  |  |  |  |  |  |
| Treatment | 41 | 7.44 (0.84) | 5.12 (0.67) | 3.58 (1.11, 6.05) | p<.001 | 6.63 (0.92) | p=.001 | p=.266 | 31% (14, 49) | 11% (-13, 35) |
| Control | 44 | 6.09 (0.69) | 6.81 (0.70) |  |  |  |  |  | -12% (-34, 11) | .. |
| **ABI** |  |  |  |  |  |  |  |  |  |  |
| Treatment | 29 | 8.17 (0.91) | 6.13 (1.00) | 1.7 (0.32, 3.07) | p=.004 | 5.55 (1.03) | p=.374 | p=.012 | 25% (1, 49) | 32% (7, 57) |
| Control | 24 | 9.08 (1.10) | 10.04 (1.19) |  |  |  |  |  | -11% (-36, 15) | .. |
| **PD** |  |  |  |  |  |  |  |  |  |  |
| Treatment | 25 | 5.00 (0.78) | 4.63 (0.86) | 0.25 (-1.62, 2.12) | p=.484 | 4.63 (0.78) | p=.988 | p=.607 | 7% (-26, 41) | 7% (-23, 38) |
| Control | 18 | 4.50 (1.19) | 4.88 (0.96) |  |  |  |  |  | -8% (-50, 33) | .. |
| **Epilepsy** |  |  |  |  |  |  |  |  |  |  |
| Treatment | 16 | 7.56 (1.43) | 5.09 (1.14) | 3.92 (1.58, 6.25) | p=.103 | 4.62 (0.93) | p=.503 | p=.023 | 33% (3, 62) | 39% (15, 63) |
| Control | 18 | 9.67 (1.28) | 8.67 (1.26) |  |  |  |  |  | 10% (-15, 36) | .. |

** Percentage improvement is the average percentage change in mean scores (e.g., pre-treatment – post-treatment/ pre-treatment).

3MFU = 3 month follow-up;

ABI = Acquired Brain Injury; brEASI = The Brief Epilepsy Anxiety-modified; CI = Confidence Intervals; MS = Multiple Sclerosis; NDDI-E= Neurological Depressive Disorders Inventory-Epilepsy; PD = Parkinon’s Disease; Post = Posttreatment;

Pre= Pre-treatment; Tx = Treatment; SE = Standard Error.
